# Radiographic features and prognosis of early- and late-onset non-small cell lung cancer immune checkpoint inhibitor-related pneumonitis

**DOI:** 10.1186/s12885-021-08353-y

**Published:** 2021-05-29

**Authors:** Aiben Huang, Yang Xu, Xuelei Zang, Chongchong Wu, Jie Gao, Xiaoli Sun, Mei Xie, Xidong Ma, Hui Deng, Jialin Song, Fangping Ren, Li Pang, Jin Qian, Zhaofeng Yu, Shiyu Wan, Yuanyuan Chen, Lei Pan, Guanglei Zhuang, Sanhong Liu, Xinying Xue

**Affiliations:** 1 Department of Respiratory and Critical Care, Beijing Shijitan Hospital, Capital Medical University, Beijing, China; 2 Department of Respiratory and Critical Care, Chinese PLA General Hospital, Beijing, China; 3 Center of Clinical Laboratory Medicine, the first Medical Centre, Chinese PLA General Hospital, Beijing, China; 4 Department of Radiology, Chinese PLA General Hospital, Beijing, China; 5 Department of Pathology, Chinese PLA General Hospital, Beijing, China; 6 Department of Radiology, Affiliated Zhongshan Hospital of Dalian University, Dalian, China; 7 Department of Respiratory Medicine, Weifang Medical University, Weifang, China; 8 School of Medicine, Peking University, Beijing, China; 9 Shanghai Key Laboratory of Gynecologic Oncology, Renji Hospital, School of Medicine, Shanghai Jiao Tong University, Shanghai, China; 10 Institute of Interdisciplinary Integrative Medicine Research, Shanghai University of Traditional Chinese Medicine, Shanghai, China

**Keywords:** Immunotherapy, NSCLC, Checkpoint inhibitor-associated pneumonia, Prognosis, Radiographic patterns

## Abstract

**Background:**

Immunotherapy is becoming a standard of care for non-small cell lung cancer (NSCLC). Checkpoint inhibitor-associated pneumonia (CIP) is a rare and potentially life-threatening event that can occur at any time during tumor immunotherapy. However, there may be differences in the radiological patterns and prognosis of CIP during different periods. This study aimed to investigate the radiographic features and prognosis of early- and late-onset immune-related pneumonitis.

**Methods:**

We retrospectively analyzed the clinical data of 677 NSCLC patients receiving immunotherapy to identify 32 patients with CIP, analyzed the clinical and radiographic data, and summarized the radiological features and prognosis of early- and late-onset CIP.

**Results:**

CIP had an incidence of 4.7%, a median onset time of 10 weeks, and a mortality of 28.1%. Among these, CIP included 14 early-onset cases, where grade ≥ 3 CIP accounted for 92.9%, main radiographic pattern was organizing pneumonia (OP)-like pattern, and mortality was 50.0%. We also identified 18 late-onset CIPs, where grade ≥ 3 CIP accounted for 50.0%, main radiographic pattern was nonspecific interstitial pneumonia (NSIP)-like pattern, and mortality was 11.1%. The overall survival rate of the early-onset group was significantly lower than that of the late-onset group (*P* < 0.05).

**Conclusion:**

Early-onset CIP cases were higher in the Common Terminology Criteria for Adverse Events (CTCAE v5.0) grade and mainly presented with an OP-like radiographic pattern; whereas, late-onset CIP cases were lower in CTCAE grade and mainly presented with an NSIP-like radiographic pattern. Finally, the prognosis of the early-onset CIP group was poorer than that of the late-onset CIP group. We believe that this study will be helpful for clinicians for making early diagnosis and deciding treatment modalities for patients with CIP.

**Supplementary Information:**

The online version contains supplementary material available at 10.1186/s12885-021-08353-y.

## Background

In the last decade, immune checkpoint inhibitors (ICIs) have played an increasingly important role in the treatment of advanced non-small cell lung cancer (NSCLC), malignant melanoma, and other malignancies [1, 2]. However, with the continuously expanding indications of ICIs and their widespread application in clinical front-line treatment, many immune-related adverse reactions associated with ICIs have been observed, for instance, in the skin, pituitary gland, thyroid gland, liver, kidney, lung, and other organs; these reactions can even be life-threatening [3, 4].

Checkpoint inhibitor-associated pneumonia (CIP) is a rare fatal immune-related adverse event (irAE), which has an incidence rate of 2–5% [5]. Nishino et al. found that the incidence of CIP is higher in NSCLC than in melanoma [6]. Further, compared with patients with other malignant tumors, NSCLC patients had a higher mortality rate associated with ICI treatment [7, 8]. CIP could occur at any time during tumor immunotherapy [9], and typically varies from 2 to 24 months [10]. Nakahama et al. [11], Erwin et al. [12], and Costa et al. [13] have found that CIP can occur within a few days after ICI application. However, the clinical manifestations of these patients were inconsistent, for instance, some patients developed respiratory failure quickly; whereas, other patients showed no obvious discomfort, even though their chest computed tomography (CT) showed an obvious exudation shadow. Erwin et al. found that patients with early CIP have severe clinical symptoms [12]. Furthermore, the radiographic pattern of CIP is complicated. In addition to the typical cryptogenic organizing pneumonia (COP)-like, nonspecific interstitial pneumonia (NSIP)-like, hypersensitivity pneumonia (HP)-like, and acute interstitial pneumonia (AIP)-like interstitial patterns, there are some uncommon radiographic patterns such as sarcoid-like granulomatosis [14] and pleural effusion [15]. However, there are no reports that evaluate the radiographic difference between early- and late-onset CIP. The aim of this study was to investigate the imaging patterns and prognosis of CIP in different stages.

## Methods

### Subjects

We retrospectively analyzed the clinical data of 677 NSCLC patients who received ICI treatment (anti-programmed death 1/programmed death ligand 1) between January 2017 and September 2020 at one of two hospitals (the General Hospital of the people’s Liberation Army and Affiliated Beijing Shijitan Hospital of Capital Medical University). A total of 32 patients with CIP were identified, and patients with infection or tumor progression were excluded. Further, patients who had received epidermal growth factor receptor-tyrosine kinase inhibitor (EGFR-TKI) were also excluded to rule out EGFR-TKI-induced interstitial lung disease (ILD). Postoperative histopathology, CT-guided lung puncture biopsy, or tracheoscopic bronchial mucosa biopsy was used to identify the 677 patients with NSCLC. Clinical data of the patients included sex, age, smoking history, underlying diseases, allergy history, tumor location, pathological pattern, degrees of differentiation, clinical stages, clinical symptoms, treatment methods, and overall survival. All patients were followed up from the time of initiation of ICI therapy to death or last research phone call by a member of the research team. The study was approved by the ethics committee of the hospital.

### CIP, peritumoral infiltration (PTI), and criteria for CIP classification

The diagnosis of CIP was determined by the treating oncologist (Mei Xie) and confirmed by a multidisciplinary irAE team consisting of a pulmonologist (Aiben Huang), radiologist (Xiaoli Sun) and a second oncologist (Chongchong Wu). For identifying CIP patients, we considered patients who developed dyspnea or other respiratory symptoms (including cough, shortness of breath, etc.) after treatment with ICIs, along with the presence of new radiographic infiltration and lack of evidence of pulmonary infection or other alternative etiologies (tumor progression, radiation pneumonitis, diffuse alveolar hemorrhage, heart failure, etc.). Briefly, using a combination of clinical (history and examination, arterial blood gas analysis, and pulmonary function testing), radiographic (presence or absence of tumor progression and pattern of parenchymal infiltrates), and biologic parameters (white blood cell count, sputum and/or bronchoalveolar lavage cultures, respiratory viral cultures, and cytopathologic testing), we excluded alternative etiologies such as heart failure, infection, and tumor progression. Improvement was defined as decrease in oxygen requirement, increase in exercise capacity, or improvement in radiographic infiltrates after commencement of CIP treatment. Conversely, worsening was defined as lack of improvement in oxygen requirement and exercise capacity after 72 h of steroid therapy [16]. CIP that occurred within 6 weeks of ICI treatment and after 6 weeks of ICI treatment were considered early- and late-onset CIP, respectively [17]. Further, PTI was defined as ground glass opacity (GGO) confined to the area around the tumor [17]. CIP was graded using version 5.0 of the Common Terminology Criteria for Adverse Events (CTCAE, v5.0), published in November 2017 by the United States National Cancer Institute, as follows: grade 1: asymptomatic, clinical or diagnostic observations only, intervention not indicated; grade 2: symptomatic, medical intervention indicated, limiting instrumental activities of daily living (ADL); grade 3: severe symptoms, limiting self-care ADL, oxygen indicated; grade 4: lift-threatening respiratory compromise, urgent intervention indicated (e.g. tracheotomy or intubation); and grade 5: death [18].

### Chest CT

In this study, all the patients underwent a non-contrast CT of the chest by the performance of a Siemens SOMATOM Sensation 64-Slice CT Scanner (Siemens, Forchheim, Germany) or a GE LightSpeed 16-Slice CT scanner (GE Healthcare, Beijing, China). CT related parameters were as follows: routine section thickness1.0, 1.25, or 1.5 mm; section thickness after reconstruction: 0.625–1.25 mm; filtered back-projection reconstruction method; 80–120 kV; 200–280 mAs; and a B70f kernel. The data of these 677 patient’s chest CT were procured from picture archiving and communication system (PACS).

### Chest CT interpretation

The chest CT images were inspected by a thoracic radiologist (J.W., with 24 year-experience in cardiopulmonary imaging) and a medical student (Y.T., with 2 year-experience in pulmonary imaging diagnosis) from each institution by using PACS (AGFA Healthcare, Mortsel, Belgium; lung window width, 1500 HU; level, 2500 HU) and traced the chest CT images that were highly suspected to be CIP in consensus. Whereafter, the presence of CIP in these patients were confirmed by a radiologist in pulmonary imaging (S.Z., with 17 year-experience in pulmonary imaging diagnosis) and a pulmonary radiologist (C.W., with 15 year-experience in pulmonary imaging diagnosis). Two radiologists mentioned above were analyzed the same patient independently on the same day, and the radiologists resolved the disagreement under discussion to reach a consensus. All radiologists were not aware of the pathologic diagnoses of the patients. The record of each lesion on the CT images included the number of involved lobes and lung area of CIP, consolidation, presence and distribution of GGO, nodularity, reticulation, pleural effusion, traction bronchiectasis, and determination of the validity of ILD in new diffuse infiltration. Radiology severity of CIP stratified into mild, moderate, and severe [18]. CT findings of ILD were ranged according to the American Thoracic Society/European Respiratory Society (ATS/ERS) international multidisciplinary classification of IP as AIP/DAD-like pattern, HP-like pattern, COP-like pattern, NSIP-like pattern, and others.

### Statistical methods

Excel (Microsoft) was used for data collection, and SPSS version 26.0 (IBM Statistics, Armonk, NY, USA) was used for statistical analysis. Numerical data are expressed as mean ± standard deviation. Chi-square or Fisher’s exact test was used for categorical data. Patient survival was estimated by Kaplan-Meier method and compared with the log-rank test. *P* < 0.05 was considered significant.

## Results

### Clinical features

**Fig. 1:**
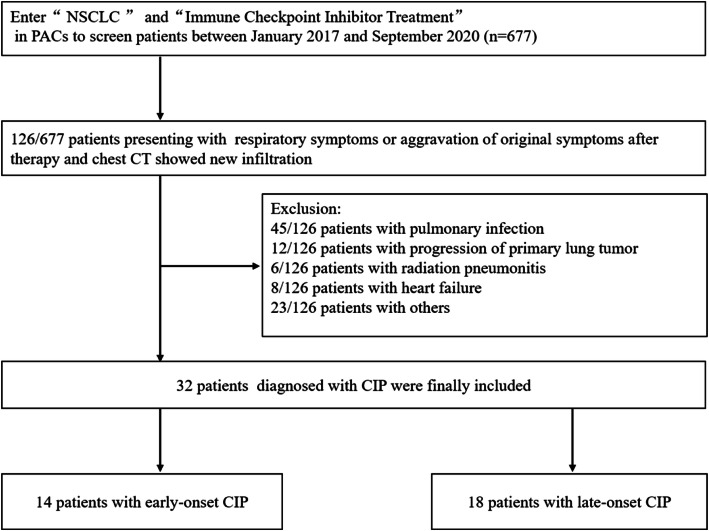
Flowchart of Screening CIP Patients

Figure 1 shows the flowchart of screening the patients. A total of 32 (4.7%) patients with CIP (mean age: 64.1 ± 10.3 years; age range: 43–82 years) were included in this study, including 26 (81.2%) males (mean age: 64.0 ± 10.3 years; age range: 43–82 years) and 6 (18.8%) females (mean age: 64.5 ± 11.3 years; age range: 45–78 years). The median onset time was 10 weeks (0.1–71 weeks), and early-onset and late-onset CIP accounted for 43.8 and 56.2%, respectively. Among these patients, 25 (78.1%) had a history of smoking, with an average of 40.4 ± 31.5 pack-years (range: 22.2–58.6 pack-years). Of the 32 patients, 21 (65.6%) patients were administered pembrolizumab, 6 (18.8%) were administered nivolumab, 2 (6.3%) were administered sintilimab, 2 (6.3%) were administered durvalumab, and 1 (3.1%) was administered nivolumab and ipilimumab. Thirty-two CIP patients received steroid treatment, after which, 23 (71.9%) patients survived, including 17 patients’ condition improved, 6 patients’ condition worsened. In addition, 9 (28.1%) patients died. Clinical features are detailed in Table 1.

**Table 1:** Baseline Characteristics of 32 CIP Patients

Characteristics	Datum
Age, years, mean ± SD	64.1 ± 10.3
Male, n (%)	26 (81.3)
Current smoking, n (%)	25 (78.1)
Symptoms, n (%)	
Cough	17 (53.1)
Dyspnea	29 (90.6)
Fever/Fatigue/Chest pain/No symptom	11 (11.3)
Tumor location, n (%)	
Left lung	13 (40.6)
Right lung	19 (59.4)
Tumor histology, n (%)	
squamous	9 (28.1)
adenocarcinoma	20 (59.4)
Squamous+ adenocarcinoma	2
others	1 (12.5)
Initial tumor stage, n (%)	
I-II	1 (3.1)
III-IV	31 (96.9)
ICI agents, n (%)	
pembrolizumab	21 (65.6)
Nivolumab	6 (18.8)
Sintilimab/durvalumab/nivolumab+ipilimumab	5 (15.6)
Surgery, n (%)	7 (21.9)
Chemotherapy, n (%)	18 (62.5)
Radiotherapy, n (%)	8 (25.0)
Radiation pneumonitis, n (%)	2 (6.3)
Prognosis, n (%)	
Survival	23 (71.9)
Death	9 (28.1)

### Radiographic patterns of CIP

**Fig. 2:**
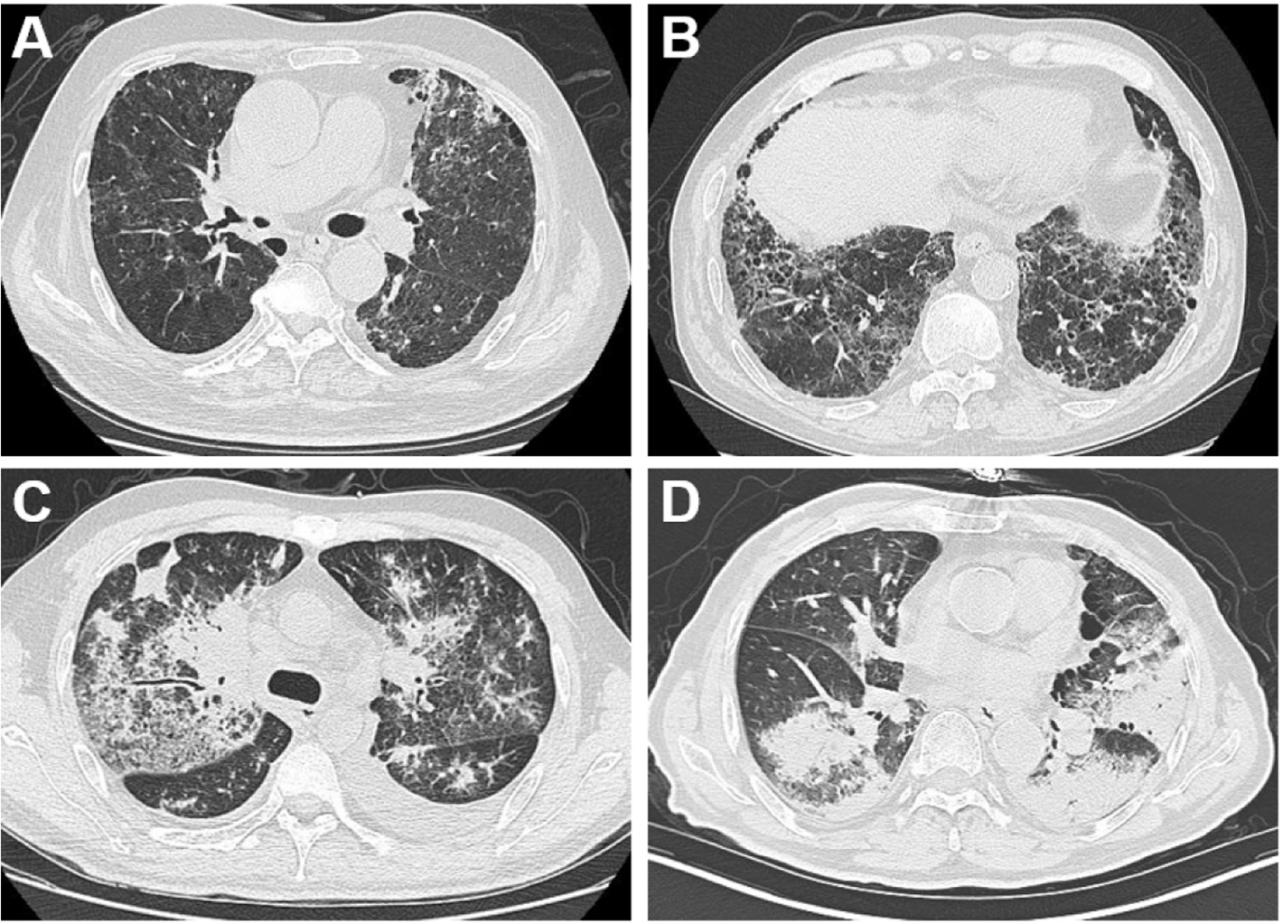
Radiographic patterns of CIP. **a** HP-like pattern: GGO and nodules observed at left lung and right upper lung. **b** NSIP-like pattern: GGO and reticulation shadows observed at bilateral lungs. **c** AIP-like pattern: Diffuse GGO observed at bilateral upper lung and bilateral lower lobe dorsal segment, accompanied by consolidation and reticulation shadows. **d** OP-like pattern: Consolidation shadow and scattered GGO observed at left lung and right subpleural lower lung

The chest CT patterns of the 32 patients with CIP included GGO (29 patients, 90.6%), reticulation (14 patients, 43.8%), consolidation (12 patients, 37.5%), nodularity (8 patients, 25.0%), bronchitis (7 patients, 21.9%), pleural effusion (2 patients; 6.3%), and among which 5 (15.6%) patients showed PTI features. The radiographic patterns of CIP included OP-like pattern (11 patients, 34.4%; Fig. 2a), NSIP-like pattern (11 patients, 34.4%; Fig. 2b), HP-like pattern (2 patients, 6.3%; Fig. 2c), and AIP-like pattern (1 patient, 3.1%; Fig. 2d).

### Difference in radiographic patterns of early-onset and late-onset CIP

According to the occurrence time of CIP after ICI treatment, the patients were categorized into early-onset and late-onset CIP groups. In the early-onset CIP group, the chest CT patterns included GGO (13 patients, 92.9%), consolidation (7 patients, 50.0%), reticulation (5 patients, 35.7%), nodularity (5 patients, 35.7%), and bronchitis (3 patients, 21.4%); whereas, in the late-onset CIP group, the chest CT patterns included GGO (16 patients, 88.9%), reticulation (9 patients, 50.0%), consolidation (5 patients, 27.8%), nodularity (3 patients, 16.7%), bronchitis (4 patients, 22.2%), and pleural effusion (2 patients, 11.1%), indicating that there was no statistical difference in the radiographic patterns between both the groups (*P* > 0.05).

The radiographic patterns of early-onset CIP included OP-like pattern (7 patients, 50.0%), NSIP-like pattern (2 patients, 14.3%), HP-like pattern (1 patient, 7.1%), AIP-like pattern (one patient, 7.1%), and others (3 patients, 21.4%); whereas, the radiographic patterns of late-onset CIP included OP-like pattern (4 patients, 22.2%), NSIP-like pattern (9 patients, 50.0%), HP-like pattern (1 patient, 5.6%), and others (4 patients, 22.2%), thereby indicating that there was no statistical difference in the radiographic patterns between both the groups (*P* > 0.05).

**Fig. 3:**
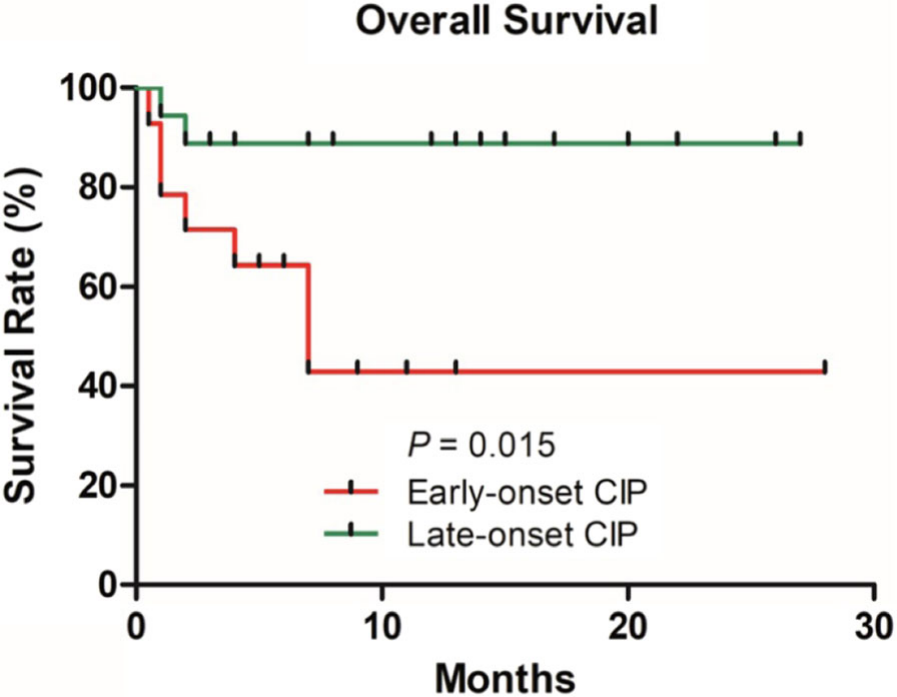
Difference in OS between early-onset and late-onset CIP groups

According to the grading system of CTCAE v5.0, there was one patient with grades 1–2 CIP (7.1%) and 13 patients with grades 3–5 CIP (92.9%) in the early-onset group. Whereas, in the late-onset group, 9 patients had grades 1–2 CIP (50.0%) and 9 patients had grades 3–5 (50.0%), statistical difference was observed in the CIP grading between both the groups (*P <* 0.05). In the early-onset group, 7 patients (50.0%) survived and 7 patients (50.0%) died; whereas, in the late-onset group, 16 patients (88.9%) survived and 2 patients (11.1%) died, indicating a statistical difference in the overall survival (OS) between both the groups (*P <* 0.05; Table 2 and Fig. 3).

**Table 2:** Radiographic patterns of 14 early-onset CIPs and 18 late-onset CIPs

	Overall *N* = 32	Early-onset CIP *N* = 14	Late-onset CIP *N* = 18	*P*
**CIP location**, n (%)				
Bilateral	25 (78.1)	10 (71.4)	15 (83.3)	0.459^*^
Left	4 (12.5)	3 (21.4)	1 (5.6)	
Right	3 (9.4)	1 (7.2)	2 (11.1)	
**Number of lobes involved**, n (%)				
1–3	10 (31.3)	5 (35.7)	7 (38.9)	0.712^*^
4–5	22 (68.7)	9 (64.3)	11 (61.1)	
**Involves area of lung parenchyma**				
≤ 50%	14 (43.8)	4 (28.6)	11 (61.1)	0.087^*^
> 50%	18 (56.2)	10 (71.4)	7 (38.9)	
**CT findings at onset of CIP**, n (%)				
Ground glass opacity	29 (90.6)	13 (92.9)	16 (88.9)	0.598^*^
Consolidation	12 (37.5)	7 (50.0)	5 (27.8)	
Reticulation	14 (43.8)	5 (35.7)	9 (50.0)	
Bronchitis/Nodularity	15 (46.9)	8 (57.1)	7 (38.9)	
Pleural effusion	2 (6.3)	0 (0)	2 (11.1)	
**Overall pattern of ILD**, n (%)				
OP-like pattern	11 (34.4)	7 (50.0)	4 (22.2)	0.096^*^
NSIP-like pattern	11 (34.4)	2 (14.3)	9 (50.0)	
AIP/HP/Others-like pattern	10 (31.3)	5 (35.7)	5 (27.8)	
**Grades of CIP**, n (%)				
1–2	10 (31.3)	1 (7.1)	9 (50.0)	0.019^*^
3–5	22 (68.7)	13 (92.9)	9 (50.0)	
**Prognosis**, n (%)				
Survival	23 (71.9)	7 (50.0)	16 (88.9)	0.022^*^
Dead	9 (28.1)	7 (50.0)	2 (11.1)	

* Fisher exact test

## Discussion

ICIs have played an increasingly important role in the treatment of advanced malignant neoplasms in various systems [19]. However, with the expansion of clinical application, irAEs, especially fatal ones such as CIP, have drawn increasing attention. Sintilimab, a fully human anti-PD-1 monoclonal antibody, has a safety profile and effectiveness consistent with other approved PD-1 antibodies, which is only approved in China and used in the treatment of classic Hodgkin’s lymphoma and NSCLC [20, 21]. Despite its infrequency, CIP shows no specificity in clinical findings but has complex radiographic patterns, which makes early diagnosis difficult. For some patients, a missed early diagnosis of CIP may have fatal consequences. Furthermore, CIP can occur at any time during the course of tumor immunotherapy. However, there is no report that determines the relationship between the occurrence time of CIP and prognosis. In this study, we found that there was a significant difference in the prognosis of early- and late-onset CIP patients. Accordingly, we proposed the concept of early-onset and late-onset CIP.

In multiple clinical trials, 3–7% of patients had CIP after treatment with ICIs for solid tumors [22], among which approximately 0.8% were grade ≥ 3 CIP [6]. In this study, we found that 4.7% of the patients had CIP, and grade ≥ 3 CIP accounted for 68.8%, which was significantly higher than the results of previous clinical trials. This difference may be associated with the patient selection in the mentioned clinical trials, where some patients with CIP high-risk factors (advanced age, complicating interstitial pneumonia, or radiation pneumonitis) were rejected. The median onset time of CIP is reportedly 2.8 months [18], and the median onset time in our study was 10 weeks (0.1–71 weeks), and early-onset and late-onset CIP accounted for 43.8 and 56.2%, respectively. In early-onset CIP, 59.1% were grade ≥ 3 CIP, indicating that severe patients often had CIP in the early stage of ICI treatment despite the low incidence of CIP. Baba et al. [23] found that the mortality rate of nivolumab-related interstitial pulmonary diseases was 17.4%. In this study, the mortality rate of CIP was 28.1%. After group assignment, it was found that the mortality of the early-onset CIP group (50.0%) was significantly higher than that of the late-onset CIP group (11.1%). The results of the log-rank test suggested that the median survival time of the early-onset CIP group was only 7 months, and the OS rate of the early-onset CIP group was significantly lower than that of the late-onset CIP group. Thus, physicians should identify high-risk patients during the early stages and perform regular follow-ups to ensure an early diagnosis and treatment.

**Fig. 4:**
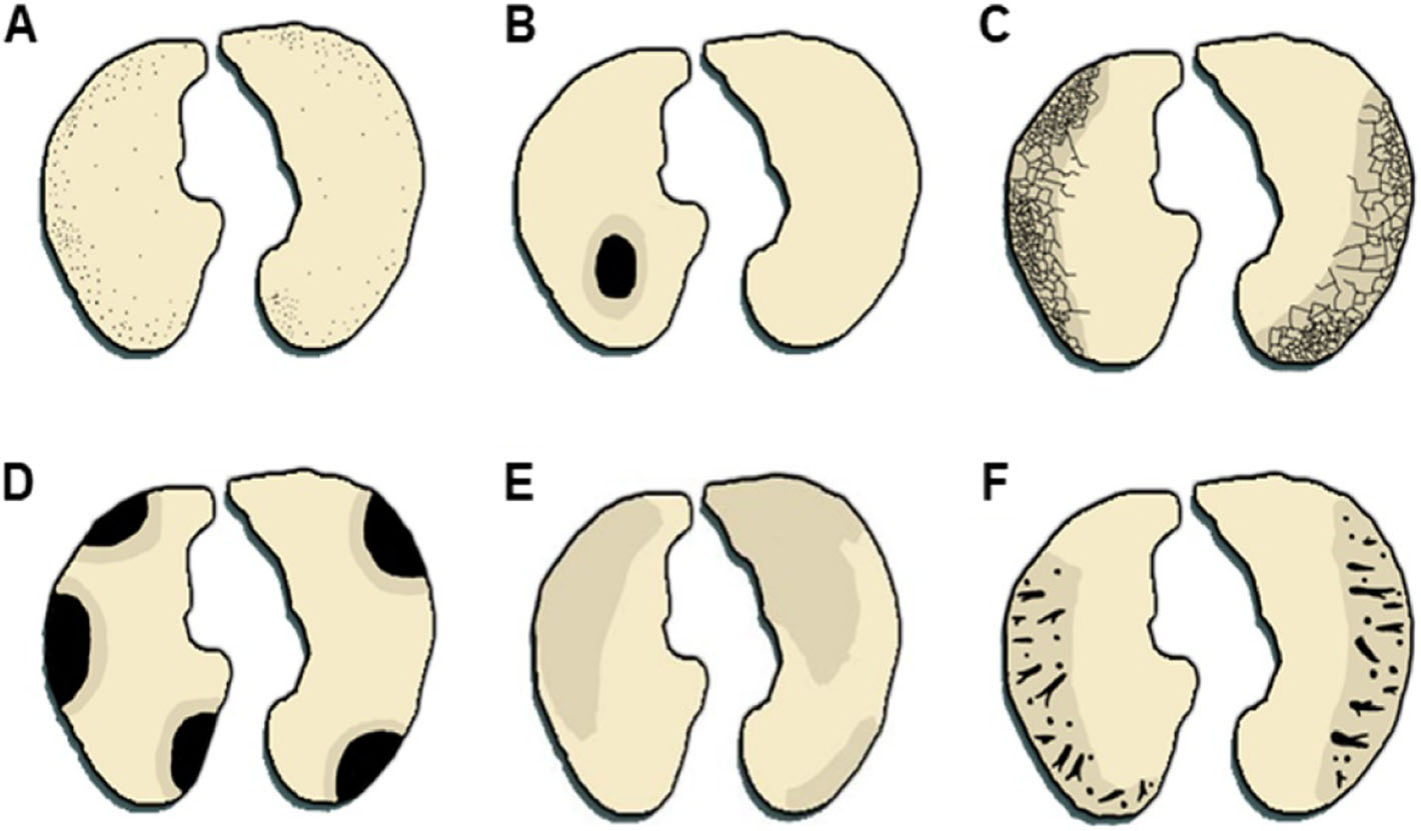
Chest CT patterns of CIP. **a** Nodularity. **b** PTI. **c** Reticulation. **d** Consolidation. **e** Ground glass opacity. **f** Bronchitis

CIP has various radiographic patterns characterized by nodularity, PTI, reticulation, consolidation, GGO, interlobular septal thickening, and funicular opacity (Fig. 4). According to the ATS/ERS international multidisciplinary classification of IP [24], the radiographic patterns of CIP are characterized by NSIP-, OP-, GGO-, and AIP-like patterns. This study found that CIP was often involved in multiple lobes and segments of both lungs, and radiographic patterns were consistent with those reported in the literature, where the most common pattern was GGO, followed by OP-like, NSIP-like, bronchitis-like, nodularity, and AIP-like patterns. Meghan Shea et al. reported that one patient had fatal CIP after treatment with pembrolizumab, and the post-onset chest CT suggested an OP-like change [25]. Nishino et al. reported that two patients receiving nivolumab treatment for advanced NSCLC had CIP in the early stages after treatment, and their chest CT suggested GGO and OP-like changes [10]. Nishino et al. also reported the chest CT findings and radiographic patterns of 20 patients with malignant tumors (10 with malignant melanoma, 6 with lymphoma, and 4 with pulmonary malignancy) who had developed CIP after treatment with immune checkpoint blockade [26]. Out of 20 patients, 7 had developed CIP in early stages (CIP onset time since treatment: 0.5–1.4 months). Their chest CT were mainly characterized by GGO, reticular opacity, and consolidation; whereas, 6 patients (85.7%) showed an OP-like pattern, 1 patient (14.3%) showed an HP-like pattern, and no patients presented with an NSIP-like pattern. Thirteen patients developed CIP in the late stage (CIP onset time since treatment: 1.6–11.5 months). Moreover, in addition to GGO, reticular opacity, and consolidation patterns, bronchodilation and centrilobular nodularity were also observed. In terms of radiographic patterns, 7 patients (53.8%) had an OP-like pattern, 3 (23.1%) patients had an NSIP-like pattern, two (15.4%) patients had an AIP-like pattern, and one (7.7%) patient had an HP-like pattern. Therefore, the main radiographic patterns in early-onset CIP was an OP-like pattern, while the NSIP-like pattern was seen more frequently in late-onset CIP patients. The results of this study were consistent with those reported in the literature, where we identified 7 (50.0%) patients with an OP-like pattern and 3 (21.4%) patients with NSIP-like pattern in the early-onset group, compared to 9 (50.0%) patients with an NSIP-like pattern and five (27.8%) patients with an OP-like pattern in the late-onset group.

What was the reason for the difference in prognosis between early-onset CIP and late-onset CIP? Firstly, there were more grade ≥ 3 CIP patients in early-onset CIP; secondly, patients with early-onset CIP had higher radiologic severity grade (71.4% v 55.6%). It is generally believed that the prognosis of COP pattern is better. But in this study, the mortality of early-onset CIP with COP pattern as the main manifestation was higher, which was related to the higher proportion of severe radiologic severity in early-onset CIP group (71.4% v 25.0%) (Table 3).

**Table 3:** Radiologic severity of 32 CIP Patients

Severity	Mild	Moderate	Severe
CT Image			
Description	Confined to one lobe of the lung or Confined to < 25% of lung parenchyma	Involves more than one lobe of the lung or Involves 25–50% of lung parenchyma	Involves all lobes of the lung or Involves > 50% of lung parenchyma
Early-onset CIP	n	n	n
COP pattern	1	1	5
NSIP pattern	0	0	2
Other patterns	0	2	3
Late-onset CIP			
COP pattern	0	3	1
NSIP pattern	0	3	6
Other patterns	0	2	3

## Conclusion

Our cohort provides a new insight into the difference of radiographic pattern and prognosis between early-onset and late-onset CIP. We have shown that early-onset CIP patients often have more severe symptoms and poorer prognosis, with an OP-like pattern as the dominant radiographic pattern. Late-onset CIP patients often have fewer symptoms and better prognosis, with NSIP-like pattern as the dominant radiographic pattern. When CIP develops in clinical settings, attention should be paid to the onset time, grading, and radiographic pattern of CIP; consequently, immediate diagnosis must be performed and treatment should be provided to improve the prognosis. The current study has several limitations, including its retrospective design, small sample size, and lack of histopathological and bronchoscopic findings. Thus, additional studies with larger sample sizes are required to confirm our results.

## Supplementary Information

**Additional file 1.**

## Data Availability

All the data generated and analyzed during this study are included in this published article.

## Abbreviations

ICI: Immune checkpoint inhibitors
NSCLC: Non-small cell lung cancer
CIP: Checkpoint inhibitor-associated pneumonia
irAE: Immune-related adverse event
CT: Computed tomography
COP: Cryptogenic organizing pneumonia
NSIP: Nonspecific interstitial pneumonia
AIP: Acute interstitial pneumonia
EGFR-TKI: Epidermal growth factor receptor-tyrosine kinase inhibitor
PTI: Peritumoral Infiltration
GGO: Ground glass opacity
NCCN: National Comprehensive Cancer Network
PACS: Picture archiving and communication system
ATS/ERS: American Thoracic Society/European Respiratory Society
